# A Review of Filovirus Work and Facilities at The Defence Science and Technology Laboratory Porton Down

**DOI:** 10.3390/v4081305

**Published:** 2012-08-17

**Authors:** Sophie J. Smither, Mark S. Lever

**Affiliations:** Biomedical Sciences Department, Defence Science and Technology Laboratory (Dstl), Porton Down, Salisbury, Wiltshire SP4 0JQ, UK

**Keywords:** Porton Down, facilities, containment, filovirus, research, aerosol, models, marmoset

## Abstract

Porton Down houses two separate sites capable of conducting high containment research on ACDP (Advisory Committee on Dangerous Pathogens) Hazard Group 4 agents: the Defence Science and Technology Laboratory (Dstl) and the Health Protection Agency (HPA), and filovirus research has been performed at Porton Down since the first Marburg virus disease outbreak in 1967. All work is conducted within primary containment either within cabinet lines (for *in vitro* work) or large rigid half-suit isolators (for *in vivo* work). There are extensive aerobiological facilities at high containment and the use of these facilities will be reported. Research at Dstl is primarily focused on assessing and quantifying the hazard, and testing the efficacy of medical countermeasures against filoviruses. Fundamental research directed to the study and understanding of the infectious and pathogenic nature of the filoviruses, particularly in aerosols, will be reported.

## 1. History

The area known as Porton Down in Wiltshire, South-West England (100 miles west of London) houses two government agencies: the Health Protection Agency (HPA) and the Defence Science and Technology Laboratory (Dstl). HPA support the NHS (National Health Service) and Department of Health, whilst Dstl is part of the UK MoD (Ministry of Defence). Both sites have their own research programs, but also work together on shared areas of interest, and both share the same origins. 

Porton Down as a government research facility was set up in 1915 to counteract the threat of gas from the Germans during the Great War. A location in the middle of Salisbury Plain in Wiltshire was chosen. After the war, the recommendation was to keep performing research, and so the organization grew; the site became known as the Microbiology Research Establishment (MRE) [1].

A virology section was established at MRE in 1953 and originally carried out poxvirus research. Between 1963 and 1964 the Arthropod-borne Virus Epidemiology Unit was set up, and focus switched to the arboviruses, with particular focus on Japanese encephalitis and the louping ill virus and other causes of viral encephalitis [1] 

MRE closed in 1979. The MoD Defence Microbiology Division formed the CDE (Chemical Defence Establishment), which later became CBDE (adding Biological to the title), and part of DERA (Defence Evaluation and Research Agency). Privatization of some parts of the company in 2001 led to the formation of the private company QineteQ, and the establishment of the MoD owned and funded Dstl, who continued with research unsuitable for privatization. When MRE closed as an MoD establishment the Public Health Laboratory Service (PHLS) took control, and the remains of the virology unit were found here. In 1979, PHLS became CAMR (Centre for Applied Microbiology Research) and the virology unit formed part of the AIDS and special pathogens group, later becoming the Special Pathogens Reference Laboratory. CAMR became part of the HPA in 2003. In the next two years, HPA will become part of Public Health England, and the future of the site at Porton Down remains unclear. 

What is now HPA (whilst it was still known as MRE and part of the MoD) played a role in the original Marburg virus outbreak; researchers at the time were asked to assist in diagnosis, and blood and necropsy material was received and tested in a variety of cell lines and laboratory animals [2,3]. The diagnostic support and testing in animals continued and was also put to use following the original Ebola virus outbreaks. The HPA site also performed some of the initial work on inactivation testing various chemicals and heat inactivation [4]. Some of the earlier pathogenesis papers and research into the new ebolaviruses came from scientists at MRE/HPA e.g., [5,6]. In 1976 an investigator at the HPA site became infected with Sudan virus after a needle stick accident with homogenized guinea-pig liver. The researcher did become ill, but recovered fully [7]. 

Today, HPAs role is mainly to prepare for public healthcare emergencies and provide epidemiological services and screening. Their research is primarily focused on the development of diagnostics. In terms of the filoviruses, HPA has a single cabinet-line laboratory and some capability for performing small-animal studies. Beyond the filoviruses, HPA does some research with Crimean-Congo haemorrhagic fever virus (CCHF) and other Hazard Group 3 and 4 viruses. The site that is now based at Dstl, Porton Down did not enter the filovirus research community until the 21st century. 

## 2. Legislation and Regulation

In order to understand the ways of working at High Containment at Dstl Porton Down, it is necessary to understand the legislation which covers the working with Hazard Group 4 pathogens. The primary legislation that covers all work in the UK is the Health and Safety at Work Act (1974). Within this is COSHH, the regulations for Control of Substances Hazardous to Health. There is then more specific legislation and guidance on working with, transporting, disposing *etc*. of dangerous pathogens. Further documentation and guidelines exist for genetically modified organisms, and for animal or environmental pathogens. The HSE (Health and Safety Executive) exists as the national independent watchdog for work-related health, safety and illness. They are an independent regulator and act in the public interest to reduce work-related death and serious injury across Great Britain’s workplaces. The HSE must be notified of, and approve all work, facilities, transport and storage of dangerous pathogens, the HSE also carry out inspections and is the body to which incidents are reported. 

The Advisory Committee on Dangerous Pathogens (ACDP) advises the HSE, Ministers and other governmental bodies on all aspects of hazards and risks to workers and others from exposure to pathogens. The ACDP produces the approved List of Biological Agents which is the official classification of biological agents into the appropriate Hazard Groups. ACDP Level 4/Hazard Group 4 pathogens are defined as Biological agents that cause severe human disease and are serious hazards to employees; are likely to spread to the community and there are usually no effective or prophylaxis or treatments available. All filoviruses fit this category. 

The HSE and ACDP have established a hierarchy of safety control measures for work carried out under COSHH. The list is one where the top level depends least on employee behaviour and the control measures are generally ‘fail-to-safe’ whereas for the control measures further down the list the safety is more dependent on employee behaviour and can be ‘fail-to-danger’ *i.e.*, if it goes wrong there could be an accident and/or exposure. The hierarchy is as follows:

1. Substitution or elimination (can you replace or reduce the hazard, use something less hazardous or a stimulant?)
2. Engineering controls (e.g., isolate the hazard in a cabinet)
3. Administrative controls (includes documented access and training)
4. Warnings (limited access and signage)
5. Personal Protective Equipment

This hierarchy, coupled with the fact that nowhere in the UK is there a working suited level 4 laboratory, meant that when the level 4 facilities at Dstl were designed, although they included the infrastructure for a suited system (chemical showers and air lines), the level 4 laboratories were set up with primary containment as the main control measure to satisfy the HSE. The HSE were more comfortable with cabinets and isolators as the primary containment as this was consistent with the working practices at Biosafety Level 3 and meant people already had experience working with these systems. Only UK scientists who have worked abroad have any kind of suit experience. Extensive validation of all aspects of the primary containment systems was required to assure the HSE that the laboratories were safe to work in e.g., protection factors, air flow and pressures, disinfection and sterilization. The facilities at Dstl are now considered a gold standard for the UK, and a valuable sovereign capability that must be maintained. Nearly a decade on, and with a number of years and experience of safe work at biosafety Level 4 having been achieved, the HSE is more willing to consider alternative containment systems. New laboratories may use the more familiar (to the US) positive pressure suit system. Establishment of a suited system of working would require adaption of facilities and extensive validation of decontamination procedures. It would also require a change in working practices and to all risk assessments, so the change will not be without considerable cost and time implications. 

## 3. Facilities

Dstl has two types of Level 4 containment laboratory; an *in vitro* laboratory consisting of a cabinet line and *in vivo*/aerobiology laboratories where the primary containment is rigid half-suit isolators. There is also a strong-room which consists of a bank of freezers for sample storage. 

The *in vitro* laboratory is used for growth and enumeration of viruses and is where assays are performed. The *in vivo*/aerobiology laboratories are for animal infections and/or aerosol studies. Each laboratory has its own autoclave for sterilisation. All laboratories run at negative pressure (>100 Pa) to the outside corridors, and can be sealed for fumigation. The rooms undergo approximately 25 air changes an hour. Both inlet and extract air is double HEPA-filtered. 

The *in vitro* laboratory went live with filoviruses in late 2005, and the *in vivo* laboratory did its first animal infection with filoviruses in 2007. The first non-human primate studies were carried out in 2009. Alongside the Level 4 laboratories, there are a series of air-lock doors to maintain the negative pressure to the laboratory, and other rooms for changing and showering (2 showers each for male and female per laboratory). The laboratories also contain an air lock for moving larger pieces of equipment in and out of the laboratory. Beyond the laboratories and associated rooms, there are two floors of air handling and engineering controls above the laboratory which supply and extract air to the rooms and cabinets and isolators. The floors above also allow some maintenance, such as changing light bulbs, and access to electrical sockets, to be performed with entrance to the laboratories not required. Below the laboratories there is a basement floor which contains the effluent treatment facility which collects and treats all liquid waste coming from the laboratories and showers. The liquid waste is heat and pressure treated before being released. 

### 3.1. *In vitro* Laboratory (Figure 1)

The single *in vitro* Level 4 laboratory at Dstl Porton Down contains a cabinet line made up of eight class III microbiological safety cabinets connected to a single L-shaped spine. All virus manipulation is performed within the individual microbiological safety cabinets through gauntlets attached to glove ports. All cabinets and the spine run at negative pressure to the room (>200 Pa), and have an air change rate of >300 air changes an hour. The spine is more negative than the cabinets connected to it, thus reducing the risk of cross contamination between individual cabinets, and allowing simultaneous work with multiple pathogens. Access to the cabinet line is via a disinfectant filled dunk tank or a pass box which can be independently fumigated/disinfected with formaldehyde vapour. The end of the cabinet line goes straight into a double-ended autoclave; access to the autoclave on the clean side is controlled to occur only if a complete autoclave cycle has been performed As well as the 8 cabinets coming off the spine, there are also within the spine a number of CO_2_ incubators for growing viruses in tissue culture. Some of the individual microbiological safety cabinets are bespoke designs to house specialist equipment. There is a cabinet that contains an ultra-centrifuge and another that contains a number of microscopes with the eye pieces on the outside of the cabinet. Also within the individual cabinets are other pieces of equipment that may be needed such as bench-top or micro-centrifuges, a real-time PCR machine and an ELISA plate reader. Equipment can be connected to an external computer via USB ports built into the cabinet walls. A trolley runs on a track up and down the spine to enable ease of movement between cabinets and incubators *etc*. The set up and validation of the cabinet line laboratory has been published [8]. 

**Figure 1:**
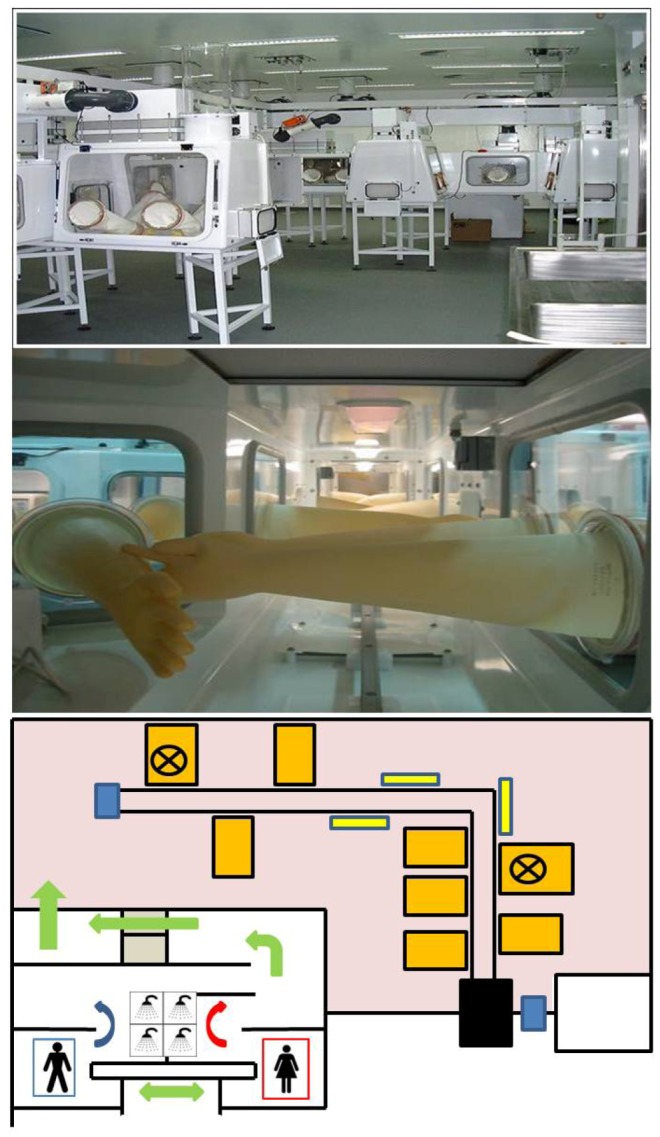
Defence Science and Technology Laboratory’s (Dstl’s) *in vitro* Level 4 Laboratory. The cabinet line with individual cabinets attached to a central spine (**top**) and a view down the central spine of the cabinet line (**middle**). A schematic of the laboratory is also shown (**bottom**) and a description given in the text: the working part of the laboratory is shaded red. Eight class III microbiological safety cabinets (orange) come off a central spine, which has a dunk tank (blue square) at one end and an autoclave (black square) at the other. Two of the cabinets contain centrifuges (a bench top and an ultra, symbolized with a circle and cross), and a number of incubators also come off the central spine (yellow boxes). Entrance to the laboratory is through a series of airlocks and change areas. The path is shown in green. There are separate change and hygiene showers for males and females. Chemical showers (grey boxes) are in place but not currently in use. As well as the autoclave there is an airlock (open box in bottom corner) and a room dunk tank to facilitate getting samples/equipment *etc*. in or out. The *in vivo*/aerobiology laboratory has the same set up except a series of isolators are in place instead of the cabinet line, and the autoclave stands alone in the room.

### 3.2. Animal/Aerobiology Laboratories

#### 3.2.1. Isolator Line and Aerosol Apparatus

The purpose of the isolator line is to allow complex animal or aerosol studies to go ahead whilst all the time maintaining an engineered form of primary containment between the pathogen and the workers. Animals are housed within the primary containment but can still be safely manipulated; all husbandry, exposures and infections, monitoring and post-mortem analysis can be performed within the isolators. The isolators are maintained at negative pressure (c. 120–150 Pa) to the room, and undergo approximately 30 air changes an hour. (This rate of 30 air changes an hour is considerably less than the 200+ in a microbiological safety cabinet, but this is for the benefit of the animals being held inside. With 30 air changes an hour, 5 min of ventilation will efficiently remove 90% circulating air and 14 min is enough to remove 99.9%). 

**Figure 2:**
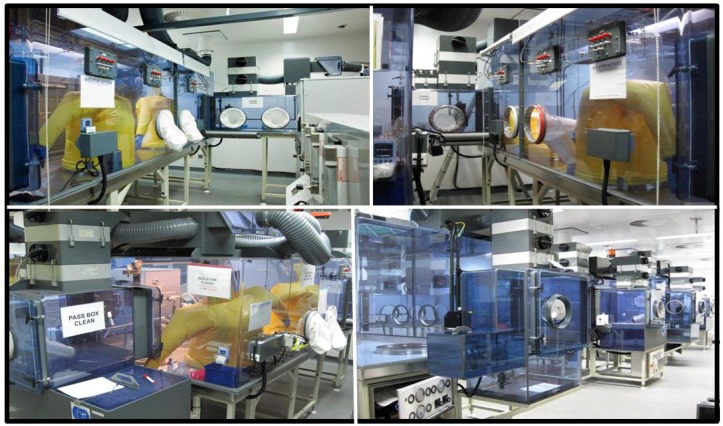
A Level 4 Animal/Aerobiology laboratory. **Top**: Views of the two ‘housing’ half-suit isolators which connect via tunnels to the middle ‘procedural’ isolator. **Top left**: Visible in the picture are marmoset cages (4) in the isolator and the Henderson apparatus for controlling aerosol exposure on the right hand side. Each isolator, pass box and tunnel has its own air supply and exhaust and can be isolated for fumigation. **Bottom left**: The procedural isolator containing the aerosol apparatus. The pass box and dunk tank for getting samples/equipment in or out are visible, as are the yellow half-suits that users work in. **Bottom right**: The isolator line during construction; view of all the pass boxes.

**Figure 3:**
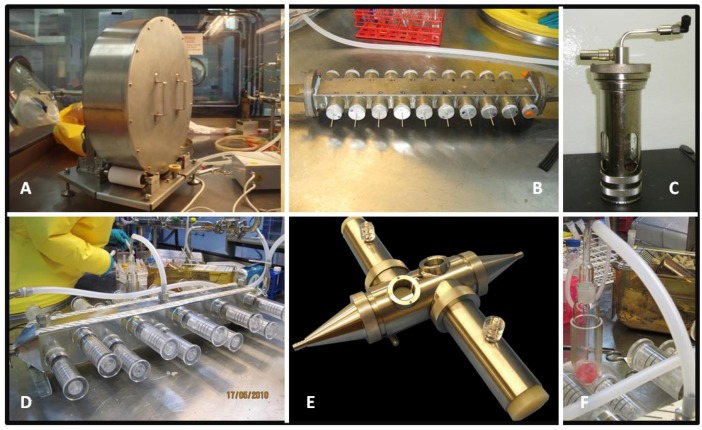
Aerosol apparatus. (**A**) 40 L Goldberg drum used for aerosol survival studies inside the isolator; (**B**) A sow used for exposing microthreads to aerosols for aerosol stability studies, in use inside an isolator; (**C**) A Collison nebuliser used to generate small particle aerosols; (**D**) Mouse exposure apparatus in use inside an isolator; (**E**) The marmoset exposure apparatus; (**F**) An AGI sampling during a mouse exposure inside an isolator.

The isolator line is made up of 3 connected isolators, connected by tunnels (Figure 2). Each isolator has two half suits and its own pass box for access. The isolators, tunnels and pass-boxes can be individually isolated and fumigated, allowing work with different species without the risk of cross contamination between isolators. Alternatively the whole line can be considered one large piece of primary containment. Two of the three isolators are designed as housing isolators and can be fitted with racks for rodent cages, or each isolator can fit four of the Dstl designed marmoset cages. Currently no work with larger primates has been performed in these isolators. Use of larger animals would require new cage design and it is estimated that it may only be possible to fit 2 to 4 animals per isolator. Alternatively, larger primate use may require the establishment of a suited laboratory similar to seen throughout the US. The third isolator is the procedural and aerosol isolator and has the necessary configurations for running aerosol experiments. The isolator can connect to either a Henderson apparatus or the BiAera Aero MP system allowing for controlled exposure to aerosolised viruses. The controls for setting up and conditioning the aerosols are located outside the isolator. The Collison nebulisers for producing aerosolised virus and all pipes and tubes are within the isolator. The Collison nebuliser is known to produce particles in the region of 1–5 µm in diameter. Aerosol sampling is through all glass impingers. (Figure 3).

The aerosol systems can connect to a variety of auxiliary apparatus, often specially designed apparatus made by the Dstl engineering department (Figure 3). Typically the aerosol apparatus connects to animal exposure units. Dstl uses specially designed nose only mouse exposure tubes, or head-only tubes for marmoset exposure (also designed to incorporate real time plethysomography during exposure, see below). Similar apparatus has been used in the past for exposing guinea-pigs. Current apparatus allows for simultaneous exposure of 20 mice or 2 marmosets. 

Alternatively, the aerosol system can be connected to other apparatus for other aerobiology work. Some examples include the use of a Goldberg drum or microthread apparatus for looking at aerosol survival, or introducing additional apparatus such as the FFAG (Flow Focused Aerosol Generator) that can produce large particle aerosols. These pieces of equipment can also be combined with animal exposure apparatus. A 40 L drum and a ‘sow’ that contains ports for 20 microthreads have been previously used in the Level 4 laboratory (see below for research using this equipment). Briefly, the ‘drum’ consists of a round sealed vessel which can be filled with an aerosol generated in the traditional way. The drum sits in a cradle with rollers that keep it slowly rotating, maintaining the aerosol inside. Sampling ports allow removal of fractions of the aerosol over time. The microthread technology uses spiders’ webs to capture aerosol droplets. Aerosol droplets will stay attached to the webs until agitated or washed off. All aerosol equipment is manufactured out of material (stainless steel) that can withstand autoclaving so that it can be safely sterilised (Figure 3).

Other equipment found in the isolators is that used routinely during animal experiments such as balances and a suite of blood analysis machines enabling sampling for complete cell counts, blood chemistries and enzymes and coagulation times (Idexx laboratories). Work using all the equipment described above is performed inside the isolators so no samples are ever manipulated outside of primary containment.

#### 3.2.2. Marmoset Cages

In the isolators the marmosets are housed in specially designed and engineered cages that offer a number of features useful for handling in maximum containment.

The cage includes a veranda which is a feature that is always popular with the pair-housed primates. It gives the animals more room to move in, and a viewing area where they can see and interact with other animals in cages next to them, and sit or play in the elevated position they prefer. The veranda can also be shut off and removed from the main cage, so can be used as a method of capturing or separating animals prior to procedures. The other important feature of the cages is the retractable ceiling. In a few minutes of preparation time, a fake ceiling with a Velcro net can be placed at the top of the cage. Handles on the outside of the cage then allow the ceiling to be lowered, gently forcing the animal towards the bottom of the cage. The animal is held to the bottom of the cage by the fabric net and can then be grabbed through the net and removed from the cage as the Velcro peels off. If the animal needs to be anaesthetised prior to handling, then a bowl with inhalational anaesthetic can be placed over the animal to subdue it prior to removing from the cage. The rest of the cage offers approximately 0.23 m^3^ of space for the animals, and has plenty of room for placing or hanging objects for environmental stimulation and enrichment for the animals. Typically, cages will contain a number of plastic boxes and buckets for sleeping or playing in, several perches and tubes or other puzzle devices containing food treats. Cages are also linked up to a CCTV system that means animals are observed remotely 24 hours a day without having to enter the room and disturb the animals. Cameras have infra-red technology so that when the room is dark, the animals can still be seen. (Animal rooms have a light cycle that includes a ‘dawn’ and ‘dusk’ period as lights automatically fade on and off). Cameras are frequently upgraded and the latest versions include the ability to remotely move the camera so it can pan around to view a larger area, and zoom in for clearer definition. The cameras allow both side on views of the cages, and also views from above into the whole body of the cage. Cameras view and record in colour, so coloured identification tags helps distinguish paired animals. Currently each isolator can fit 4 of the bespoke cages, meaning 8 animals can be held per isolator and 32 in total can be housed at Containment level 4 at any one time. 

#### 3.2.3. Telemetry System

**Figure 4:**
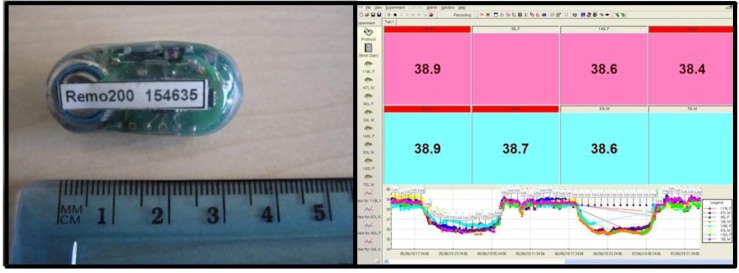
Telemetry System. Temperature implants (Remo Technologies) (**left**) are surgically implanted intra peritoneally into animals prior to a study commencing. The implants give real time core body temperature readings, updated every 30 seconds. The data is viewed (**right**) and collected in eDacq software and can be analysed in Microsoft Excel. Implants can be read when animals are pair/multiply housed. Implants can be upgraded with extra attachments, and further surgery can allow for additional readings such as hear rate or ECG *etc*.

The telemetry system used is that of Remo technologies. Remo200 temperature implants are surgically implanted into the peritoneal cavity of the animal. The Remo system allows telemetry output from multiple devices in a single cage, so animals do not have to be singly housed to receive data. All our animals are family and group housed till weaning and then pair housed from then onwards. Typically the pairs are a female and vasectomised male, but two males or two females can be housed together. The Remo200 implant gives a live real time output every 30 seconds, and is monitored through eDacq software (compatible with Microsoft Excel) (Figure 4). Telemetry can be recorded continuously, and also recorded in block periods to make data handling easier (typically raw data and hour blocks of data are generated). Data output includes the minimum and maximum temperature as well as the average for each period. Upgrades to the telemetry system will include the ability to measure further parameters as well as temperature, such as hear rate or ECG (electrocardiogram). These devices will still work when multiple animals are housed together in a small area, such as within an isolator. 

#### 3.2.4. Plethysomography

**Figure 5:**
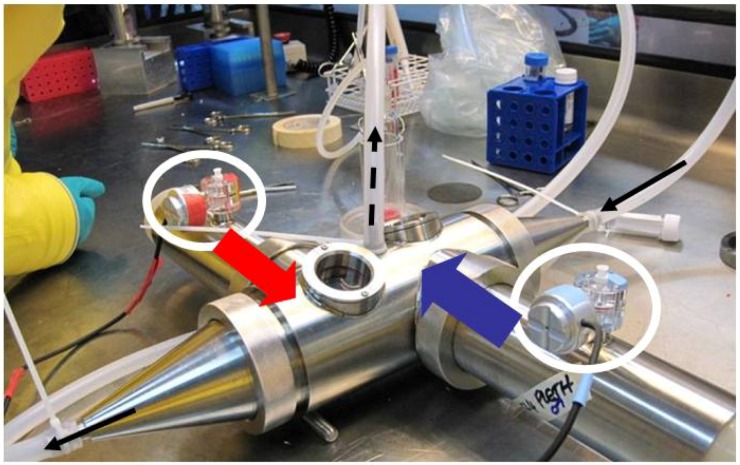
Plethysomography. The custom designed exposure and plethysmography apparatus to expose marmosets to aerosolised virus. Anaesthetised animals are placed in the tubes (coloured arrows) for head-only exposure. Fleisch pneumotacographs (circled) are attached to the tubes to allow real-time whole body plethysomography recordings during exposure. The aerosol is generated using a Collison nebuliser (partially visible in top left corner) and conditioned with the Henderson apparatus or BiAera MP system. The path of the aerosol is indicated with black arrows. During exposure, aerosol is sampled into an all-glass impinger (dashed arrow). Enumeration of impinger samples, total volume of air inhaled during challenge and flow-rate of the system enable enumeration of retained dose. All equipment is held in a rigid half suit isolator, within the Containment Level 4 laboratory.

Bespoke exposure tubes have been designed in conjunction with EMMS Ltd (Electro-Medical Measurement Systems) to allow real time plethysomography of marmosets during aerosol exposure at Containment Level 4 (Figure 5). Anaesthetised animals are inserted into tubes, and their heads only are exposed to the aerosols. Animals can be viewed through window in the body of the exposure apparatus. Connected to the tube is a Fleisch pneumotacograph that connects to an externally located transducer. The signals can be viewed using eDacq software and allow monitoring and recording of multiple parameters such as breathing rate and frequency, tidal volume and cumulative volume over a timed period. The volume of air for each individual animal is recorded, and combined with the impinger information and flow rate *etc*. of the aerosol apparatus, allows for accurate determination of the dose inhaled by individual animals. 

## 4. Research

Since 2005 the filovirus research at Dstl has broadly fallen in to two categories; understanding the threat and protection against the threat. 

Understanding the threat has been concerned with characterising the survival of the most pathogenic filoviruses (Marburg virus and Ebola virus), initially on substrates and then within aerosols, and also assessing the effect of passage and growth in different cell types on virulence of Ebola virus. Survival on plastic and glass substrates and in tissue culture media and blood has been reported [8], and as expected for enveloped viruses, the filoviruses survive quite well, particularly at lower temperatures. Survival and stability in aerosols has been studied by two different methods. Initially survival was determined as a dynamic aerosol in a rotating drum, with impinger samples taken over time. The results from these studies showed that Ebola virus and Marburg virus were both quite aero-stable with biological decay rates of approximately 4 %min^−1^ [9]. The only previous data reporting survival of Marburg virus suggested a much higher decay rate of 11.5 % min^−1^. A second method of determining aerosol survival was employed using microthreads in the forms of spiders’ webs. This methodology, based on survival of filoviruses in captured aerosols, was described recently [10]. The output gave a revised decay rate for both viruses of <3.5% min^−1^. The microthread technology is more adaptable and will likely be used in the future to test the effect of various environmental parameters (e.g., temperature, humidity or [simulated] sunlight) on survival in aerosols. The output from these experiments can be used by modellers to estimate the impact of a release (deliberate or accidental) of filoviruses. 

The other arm of research performed at Dstl is concerned with testing of medical countermeasures. To do this, appropriate animal models are needed, and Dstl has developed two animal models that supplement the existing animal models available at other institutes. The small animal model developed by Dstl is based on using wild-type virus, as opposed to adapted virus, in susceptible mice. A129 Interferon alpha/beta receptor knockout mice are the susceptible hosts of choice. These animals show susceptibility to Ebola virus (E718), Marburg virus (Popp) and Sudan virus (Boniface) by both an injected and the aerosol route of infection [11]. The mice, like humans, do not succumb to infection with Reston virus or Taï Forest virus which suggests they may be a good model for human infection in terms of susceptibility to wild-type virus. Work is underway to characterise the immune response in these mice. This mouse model shows typical liver and spleen pathology when infected with the pathogenic filoviruses and could be considered as a screening mechanism for protective compounds before down-selecting and moving into larger animals/primates. The mouse model may also be appropriate where larger numbers are needed for efficacy testing of products moving towards licensure. To date a number of pre or post exposure therapies have been tested in this mouse model. The efficacy of adenovirus expressing GP or its derivatives has been tested as a vaccine [12]. Polyclonal and monoclonal antibodies alone or in combination, heat-killed virus preparations, and small molecule inhibitors have also been tested for protective efficacy (results not shown). Protection was observed with vaccine against an injected challenge of Ebola virus, and with a monoclonal antibody combination against aerosol challenge of Ebola virus (results not shown). 

Dstl has also developed a non-human primate model of aerosolised Marburg virus infection, in the common marmoset (*Callithrix jacchus*) [13], and work is underway to develop models of Ebola virus infection by both the aerosol and an injected route to model both natural and un-natural exposure methods. Marburg virus infection resulted in a consistent temperature profile with fever evident on the 5^th^ day post infection, and the average time to death was 9 days. Inhaled doses of as low as 4 TCID_50_ units were lethal, and the pathogenesis of disease, blood parameters and histology was consistent with what is seen in other non-human primates, and also similar to the limited information gathered from human cases of filovirus infection [13]. The marmoset model offers both the advantages of a small animal model (greater capacity, quicker throughput, safer handling) and the advantages of a primate model (similarities to human disease) and so will be a useful tool in the testing and licencing of medical countermeasures against filovirus infection. 
